# Covered Endovascular Reconstruction of the Anonymous Confluence (CERAC): a technical note describing a confluence-preserving endovascular technique

**DOI:** 10.1186/s42155-026-00765-w

**Published:** 2026-09-15

**Authors:** Riccardo Corti, Ludovico Ambrosi, Nicola Cionfoli, Mauro Antonio D’Agostino, Pietro Quaretti, Antonio Bozzani

**Affiliations:** 1 Interventional Radiology Unit, IRCCS Policlinico San Matteo, Viale Camillo Golgi 19, Pavia, PV 27100 Italy; 2 Vascular and Endovascular Surgery Unit, IRCCS Policlinico San Matteo, Viale Camillo Golgi 19, Pavia, PV 27100 Italy

**Keywords:** Superior vena cava syndrome, Central venous obstruction, Stent-graft, Brachiocephalic vein, Endovascular reconstruction

## Abstract

**Introduction:**

Endovascular therapy is the first-line treatment for superior vena cava syndrome (SVCS), yet the optimal strategy for lesions involving the brachiocephalic vein confluence remains debated. We describe a technique for anatomical bilateral reconstruction of the anonymous confluence using covered stents, called Covered Endovascular Reconstruction of the Anonymous Confluence (CERAC).

**Materials and methods:**

All patients treated for SVCS between September 2024 and December 2025 were reviewed. CERAC was selectively applied based on anatomical and clinical considerations. Procedural characteristics, technical success, complications, and follow-up patency were assessed.

**Results:**

Three out of ten patients who underwent endovascular treatment for SVCS at our institution were treated using the CERAC technique. Indications included preservation of bilateral venous access in young patients with potentially treatable malignancy (*n* = 2) and emergency central venous decompression (*n* = 1). Technical success was achieved in all cases, with restoration of patency of both brachiocephalic veins and the superior vena cava. Mean fluoroscopy time was 30.6 min. No immediate complications occurred. Primary stent patency was maintained during available follow-up (29–301 days; median 149 days).

**Discussion:**

CERAC enables confluence-preserving bilateral reconstruction while minimizing stent overlap and maintaining favourable luminal geometry. Inspired by the Covered Endovascular Reconstruction of the Aortic Bifurcation, CERAC represents, to our knowledge, the first description of a fully covered, confluence-preserving reconstruction in this anatomical setting.

**Conclusion:**

CERAC is a feasible endovascular option for selected SVCS cases involving the brachiocephalic confluence. Further studies are needed to evaluate long-term outcomes and to better define appropriate indications.

**Level of evidence:**

Level V.

**Graphical Abstract:**

**Figure:**
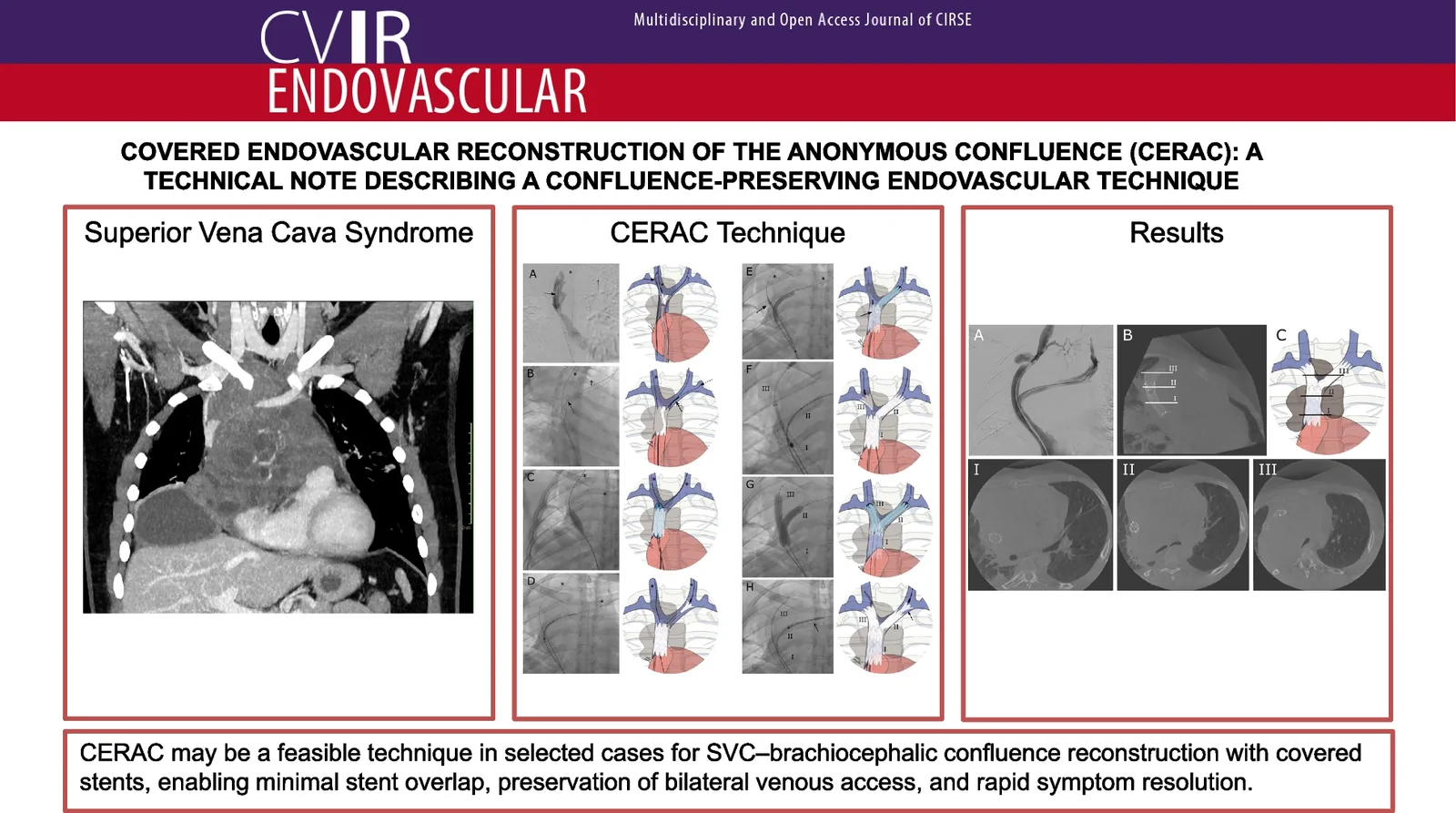

**Supplementary Information:**

The online version contains supplementary material available at https://doi.org/10.1186/s42155-026-00765-w.

## Introduction

Superior vena cava syndrome (SVCS) results from obstruction of venous inflow to the superior vena cava (SVC) and may lead to significant morbidity if left untreated.

While malignant etiologies remain common [1], the incidence of benign SVCS has increased in recent years, largely due to device-related thrombosis associated with long-term central venous access [2]. In these patients, often younger and with longer life expectancy [3, 4], durable venous patency and preservation of future vascular access are particularly important [5].

Endovascular therapy is currently considered the first-line treatment for SVCS [6], yet the optimal approach remains debated. Most studies favour unilateral stenting due to acceptable clinical relief and lower complication rates [7] while bilateral stenting may be considered in selected cases involving the brachiocephalic confluence and the SVC (Stanford and Doty III/IV), or when the SVC diameter exceeds 15 mm [8].

Several bilateral approaches have been described to address such cases, each with specific limitations. Kissing/double-barrel configurations create two parallel barrels within the SVC, with incomplete circumferential apposition predisposing to peri-stent leakage [9] and turbulent flow at the midline gap with an increased risk of premature occlusion [10–12]. Y-stenting requires passage of the branch limb through the struts of the cava stent and is therefore structurally incompatible with a covered cava stent [13]. The “train-track” technique achieves anatomical reconstruction of the confluence but with bare stents, and its authors explicitly acknowledge specific technical limitations (need for buddy-wire dilatation; impossibility of using stents or balloons with a long-tapered tip) [14].

Herein, we propose a novel endovascular technique for bilateral reconstruction of the SVC and brachiocephalic veins, the Covered Endovascular Reconstruction of the Anonymous Confluence (CERAC). This technique is designed to achieve anatomically accurate confluence reconstruction using covered stents, with the aim of preserving bilateral venous inflow while minimizing stent overlap. Conceptually inspired by the Covered Endovascular Reconstruction of the Aortic Bifurcation (CERAB) [15], CERAC represents, to our knowledge, the first description of a fully covered, confluence-preserving reconstruction of the SVC–brachiocephalic confluence.

## Materials and methods

This study is presented as a technical note (Level V evidence). All patients treated for SVCS between September 2024 and December 2025 at our institution were reviewed. In all cases, pre-procedural contrast-enhanced CT was performed to assess the extent of venous obstruction and involvement of the brachiocephalic vein confluence. CERAC was selectively applied in patients with (a) symptomatic SVC syndrome with imaging-confirmed obstruction involving both the SVC and the brachiocephalic confluence (Stanford type III/IV); (b) clinical need to preserve bilateral upper-extremity venous access (e.g. young patients, potentially treatable malignancy, anticipated need for central venous accesses); or (c) emergency indication for rapid central venous decompression in which unilateral reconstruction was judged insufficient. Patients with poor prognosis in whom the primary aim was palliation, and cases with brachiocephalic vein involvement in the absence of SVC involvement, were excluded. All CERAC procedures were performed under fluoroscopic guidance with standard monitoring. Post-procedural follow-up was conducted according to institutional protocols.

### Technique

**Fig. 1:**
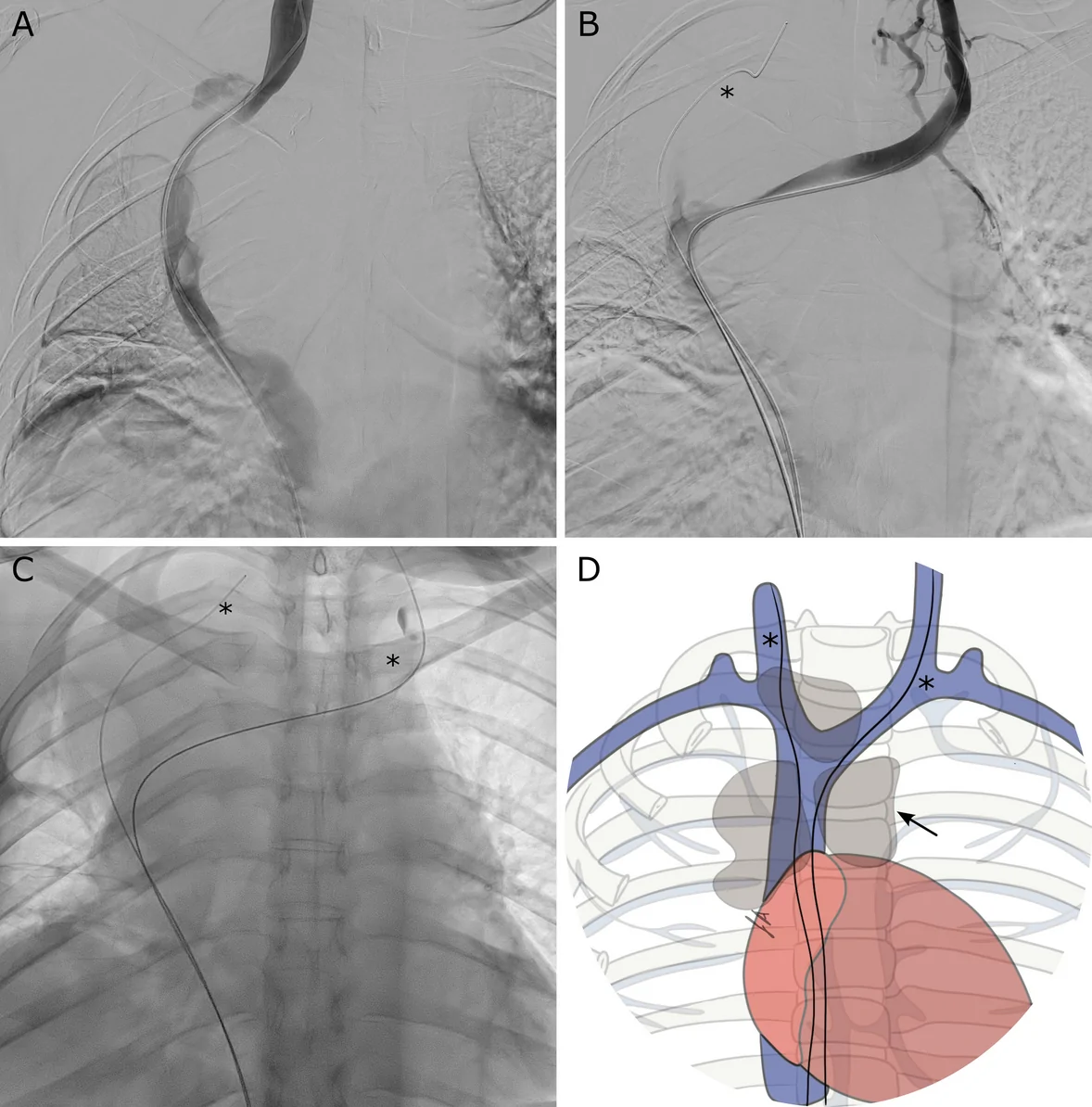
Initial venography and bilateral stiff guidewire positioning. **A**, **B** Subtracted angiograms showing stenosis of the right and left brachiocephalic veins. **C** Fluoroscopic image demonstrating bilateral stiff guidewire (*) placement. **D** Schematic illustration depicting stiff guidewire positioning (*) in both brachiocephalic veins. The *arrow* schematically represents the tumoral mass

As an illustrative example, we present the case of a 26-year-old male with a mixed germ cell mediastinal tumour, who presented with facial and chest oedema, along with right arm swelling. CT imaging revealed a large anterior mediastinal mass encasing the brachiocephalic veins and compressing the SVC (see ESM*-*Fig. 1). To preserve both venous pathways, the CERAC technique was employed.

Antibiotic prophylaxis and 5000 IU of unfractionated heparin are administered at the procedure’s outset, followed by prophylactic anticoagulation (low-molecular-weight heparin) for 2 weeks post-procedure. No antiplatelet therapy is routinely administered, given the unproven role of antiplatelets in the venous circulation, the large stent diameters employed under physiologically brisk venous flow, and the inner luminal layer of the device, engineered to reduce luminal thrombogenicity per manufacturer specifications [16, 17], as supported by independent preclinical evidence [18].

The procedure was performed under local anaesthesia and conscious sedation (midazolam), with continuous electrocardiographic and pulse oximetry monitoring.

A single US-guided femoral vein access (6 Fr 23 cm long sheath, Radifocus Introducer II; Terumo Corp., Tokyo, JP) is established. A 0.035″ hydrophilic guidewire and catheter are used to navigate through stenotic or occluded segments. Once crossed, venography is performed to delineate vascular anatomy and lesion characteristics (Fig. 1a, b).

The hydrophilic guidewire is then exchanged for a stiff guidewire (Amplatz-Superstiff; Boston Scientific International, Watertown, MA), and an 18 Fr 65 cm long sheath (Gore DrySeal; W. L. Gore & Associates, Flagstaff, AZ) is introduced to accommodate covered stent systems. The contralateral brachiocephalic vein is then accessed using the same steps until two stiff guidewires are positioned within both the stenotic brachiocephalic veins (Fig. 1c, d).

**Fig. 2:**
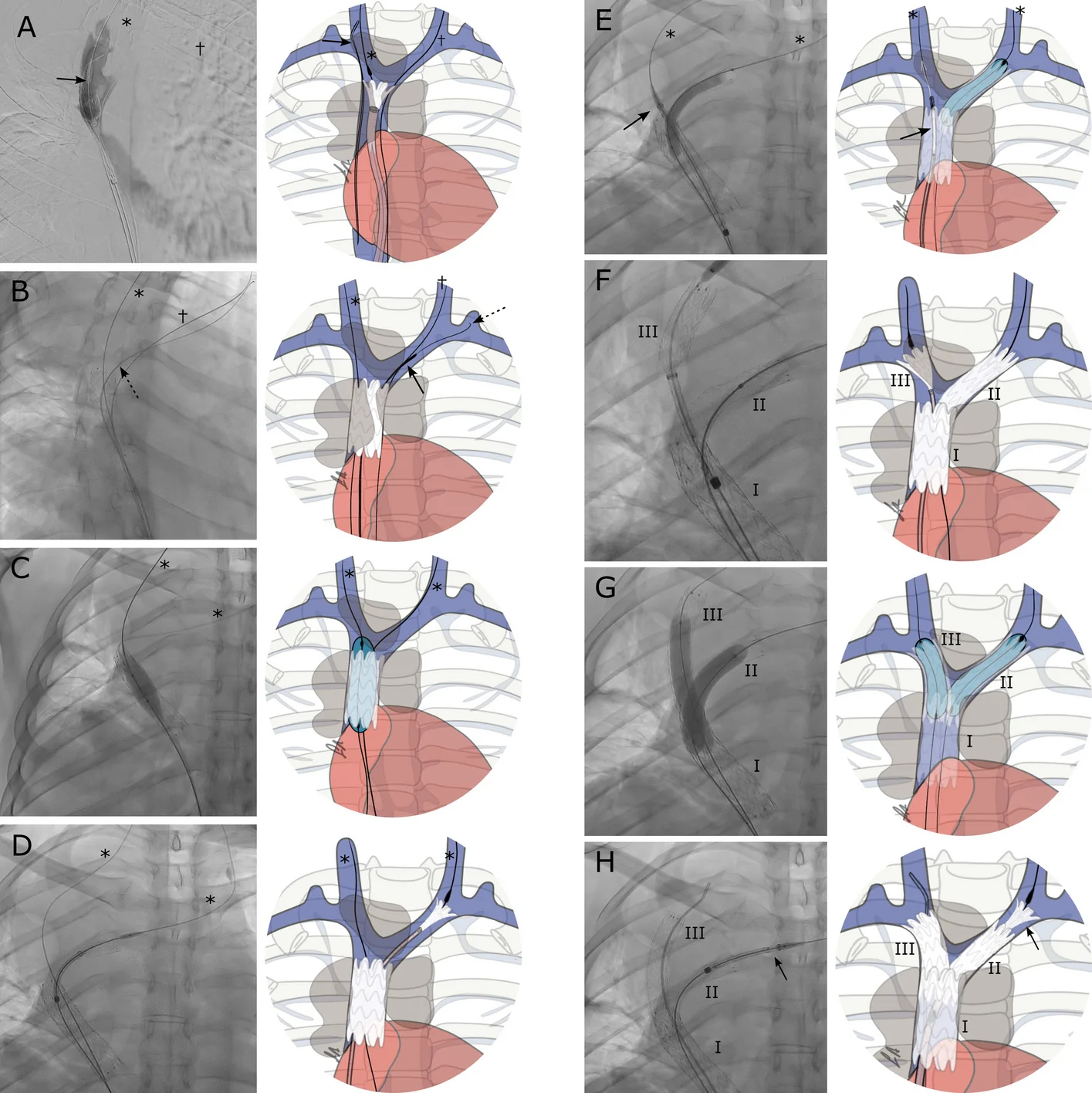
Endovascular reconstruction of the superior vena cava (SVC) and brachiocephalic veins using covered stents. **A** Digital subtraction angiography and schematic showing deployment of a 16-mm covered stent in the SVC over a stiff guidewire (*); the † symbol marks the jailed stiff guidewire, and the arrow indicates the hydrophilic catheter used for positioning. **B** Reaccess of the left brachiocephalic vein through the caval stent with a hydrophilic guidewire (dashed arrow) and catheter (arrow), while the jailed stiff guidewire (†) is maintained as a buddy wire. **C** Post-dilation of the caval stent. **D** Advancement and deployment of the first 12-mm covered stent in the left brachiocephalic vein. **E** Advancement of the second 12-mm covered stent (arrow) into the contralateral brachiocephalic vein while the balloon in the first stent remains inflated. **F** Deployment of the second 12-mm covered stent in the right brachiocephalic vein after balloon deflation. **G** Simultaneous post-dilation of both brachiocephalic stents using the kissing balloon technique. **H** Optional distal elongation (arrow) of the left brachiocephalic stent

The first step in stent deployment involves placing a 16-mm covered stent (Merit Medical Systems, Inc., South Jordan, UT) in the SVC over one of the two stiff guidewires. The stent should extend from just below the brachiocephalic confluence to the proximal SVC, ensuring complete coverage of the obstructed segment (Fig. 2a).

Before removing the stiff guidewire jailed between the caval stent and the vessel wall, the ipsilateral brachiocephalic vein is selectively reaccessed through the caval stent using a hydrophilic guidewire and catheter (Fig. 2b). Once reaccess is successfully achieved, the jailed stiff guidewire is carefully removed, and the hydrophilic guidewire in the brachiocephalic vein is exchanged for the stiff guidewire.

To optimize sealing and expansion of the caval stent, post-dilation is performed using a balloon sized to match or slightly undersized relative to the nominal stent diameter (Fig. 2c).

**Fig. 3:**
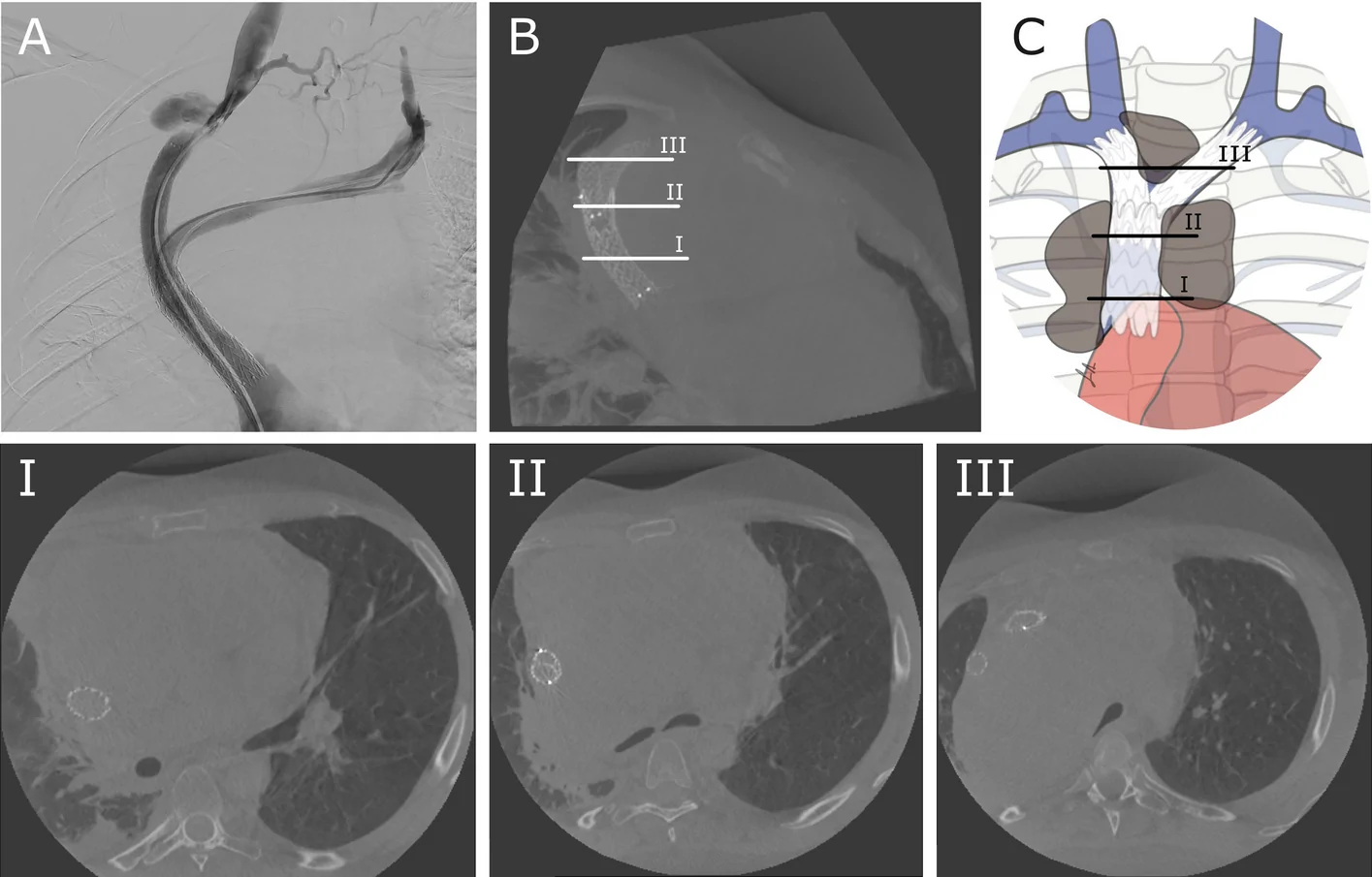
Post-procedural venography and Cone Beam CT evaluation of stent configuration. **A** Post-procedural venography confirming patency of both brachiocephalic veins and the superior vena cava (SVC) after CERAC stent reconstruction. **B** Coronal CBCT image and corresponding schematic (**C**) illustrating the spatial configuration of the SVC and brachiocephalic stents, with three anatomical levels (I–III) annotated. Level I shows the caudal segment of the caval stent within the SVC. Level II demonstrates the “double D” configuration created by the caudal ends of the brachiocephalic stents, designed to minimize excluded space and preserve a larger lumen. Level III shows the cranial portions of both brachiocephalic stents and their proximal expansion

Subsequently, two 12-mm covered stents are deployed in the brachiocephalic veins. The first stent is advanced and released in one brachiocephalic vein and post-dilated (Fig. 2d). While the angioplasty balloon remains inflated, the second covered stent is advanced into the contralateral brachiocephalic vein to avoid impingement or displacement of the first stent during positioning (Fig. 2e). After balloon deflation, the second stent is deployed (Figs. 2f and 3f), and both stents are post-dilated using the kissing balloon technique (Fig. 2g). Each brachiocephalic stent is positioned to extend approximately 1 cm into the SVC stent to minimize overlap while maintaining complete coverage; distal elongation can be performed if required (Fig. 2h). This configuration creates a “double D”-shaped lumen at the level of the brachiocephalic confluence, in which the caudal ends of the brachiocephalic stents are flared within the larger SVC stent (Fig. 3, II).

Final venography is performed to confirm patency of both brachiocephalic veins and the SVC (Fig. 3a). A post-procedural cone-beam CT (CBCT) can be obtained to assess stent apposition and spatial configuration at different anatomical levels (Fig. 3b, c).

Haemostasis is achieved via a purse-string suture.

## Results

Between September 2024 and December 2025, 10 patients with SVCS underwent endovascular treatment at our institution. The CERAC technique was selectively applied in three patients based on clinical and anatomical considerations. In two young patients, CERAC was chosen to preserve bilateral upper-extremity venous access in the setting of potentially treatable malignant mediastinal disease. In one patient, CERAC was performed in an emergency setting to achieve rapid central venous decompression due to severe upper-airway oedema, which prevented safe removal of the tracheal cannula. In the remaining patients, unilateral endovascular reconstruction was performed.

Patient and procedural characteristics of CERAC cases are summarized in Table 1. Mean fluoroscopic time was 30.6 min. Final angiography demonstrated restoration of central venous patency in all patients, with no immediate procedural complications. All patients experienced rapid clinical improvement with resolution of venous congestion symptoms. Primary stent patency was maintained throughout the available follow-up, ranging from 29 to 301 days (median 149 days).

**Table 1:** Patient and procedural details

ID	Age	Diagnosis	Symptoms	Stents Used	DAP tot [Gy cm^2^]	Fluoroscopic time [minutes]	*Primary patency [days]
1	26	Extragonadal germ-cell tumour	Sensation of “fullness” in the chest, monolateral oedema (left pectoral), and mild soreness in the left arm	4 Stents: 16 × 80 mm (SVC), 10 × 75 mm (left brachiocephalic), 12 × 80 mm (right brachiocephalic), and 12 × 40 mm (left brachiocephalic elongation)	493	29	149
2	28	Ewing sarcoma	Mantle oedema (neck/trunk swelling), jugular distension, superficial collateral circulation, dysphagia, and dyspnea	3 Stents: 16 × 60 mm (SVC), 14 × 60 mm (right brachiocephalic), and 12 × 80 mm (left brachiocephalic)	148	35	301
3	79	Neuroendocrine carcinoma	Bilateral face/limb oedema, severe oedema of the glottis and base of the tongue, cough, and dyspnoea	4 Stents: 16 × 60 mm (SVC), 12 × 80 mm (left brachiocephalic), 12 × 80 mm (right brachiocephalic), and 16 × 40 mm (SVC elongation)	421	28	29

^*^Computed using the most recent available CT scan

## Discussion

The optimal endovascular strategy for SVCS involving the brachiocephalic vein confluence remains debated, with limited comparative data between unilateral and bilateral approaches. Available studies suggest higher complication rates or reduced patency with bilateral stenting, although without definitive statistical significance [7, 19].

Previously described bilateral techniques, kissing/double-barrel, Y-stenting and the train-track approach, each entail specific mechanical, haemodynamic and procedural limitations, summarized in Table 2. Briefly, the parallel-stent geometry of kissing/double-barrel configurations is intrinsically associated with incomplete circumferential apposition to the SVC wall, peri-stent leakage and turbulent flow at the midline gap, with stagnant zones predisposing to thrombosis [9, 11, 12, 14, 15]; the combined radial tension of two large stents on the thin SVC wall has been associated with intra-procedural and delayed SVC perforation leading to fatal cardiac tamponade [9, 20, 21], a clinically meaningful concern, as SVC rupture is the leading cause of peri-procedural mortality in this population [22, 23]. Y-stenting requires the branch limb to pass through the struts of the cava stent and is therefore structurally incompatible with a covered cava stent [13]; the “train-track” technique achieves anatomical reconstruction but using bare stents [14], with the inherent limitation of tumour ingrowth between the stent struts (the dominant failure mode of bare central venous stents, reported in up to ~ 40% of malignant SVC stenting cases [24]) and some recognized technical limitations as stated by the authors.

**Table 2:** Comparison of endovascular techniques for bilateral reconstruction of the SVC–brachiocephalic confluence

Feature	Kissing/double-barrel	Y-stenting	Train-track
Stent type	Two parallel stents (bare or covered)	Bare cava stent required (branch limb crosses cava struts)	Bare stents
Confluence geometry	Two parallel “barrels” within the SVC	Branch limb passes through struts of the cava stent	Single SVC stent deployed over two wires; bare branch stents
Circumferential SVC wall apposition	Variable	Variable	Complete
Mutual stent compression/metallic “neocarina”	Yes — frequent; may cause under expansion and residual stenosis [7, 14]	In general, avoided	In general, avoided
Peri-stent leakage	High — incomplete apposition, carina sub-optimally covered [9, 14]	Low	Low
Risk of catastrophic SVC injury/tamponade	Documented, including fatal cases [9, 20, 21]; SVC rupture is the leading cause of peri-procedural mortality [22, 23]	Possible but not documented in the literature	Documented [14]
Compatibility with a covered cava stent	Yes (kissing covered stents)	No — branch limb must traverse cava-stent struts [13]	No (as described) [14]
Tumour ingrowth	Frequent with bare stents [24]; mitigated in the covered variant	Frequent (bare stents) [24]	Frequent with bare stents [24]
Specific technical/structural caveats	The stents, sized on the brachiocephalic veins, may not match appropriately the SVC diameter	Mechanical weakening of one stent due to intramesh deployment of the second brachiocephalic stent; branch-limb stability [13]	Buddy-wire dilatation required; no use of long-tapered-tip devices [14]

*SVC *superior vena cava*.* The comparison is intended to be descriptive of published features and limitations, not of demonstrated clinical superiority

The CERAC technique was developed to address these limitations by enabling anatomically accurate reconstruction of the superior vena cava–brachiocephalic vein confluence using covered stents, while preserving bilateral venous inflow and minimizing stent interaction.

Coverage of the azygos ostium, although in principle to be avoided, is acceptable in this anatomical setting: in patients with extensive central venous obstruction involving the brachiocephalic confluence and the SVC (Stanford type III–IV) the azygos system is typically already non-functional as a collateral or working in retrograde direction towards the inferior vena cava [2, 25], and once antegrade SVC flow is restored its collateral role becomes physiologically redundant. Consistently, no clinically relevant adverse event attributable to azygos jailing has been reported in published series of unilateral or bilateral covered SVC stenting [9, 24, 26, 27], and none of our patients developed signs or symptoms attributable to occlusion of the azygos vein during the available follow-up.

This technique may be particularly relevant in selected patients, such as younger individuals or those with potentially treatable malignancies, and in urgent scenarios requiring rapid central venous decompression.

In patients with potentially treatable malignant disease as well as in benign SVCS, the use of covered stents is supported by their established advantage in long-term primary patency over bare devices [26–29], primarily through abolition of tumour ingrowth. Once correctly sized and post-dilated, the reconstructed venous axis may provide a relatively stable mechanical scaffold that is less dependent on the surrounding tumour burden. Consequently, subsequent reduction in extrinsic compression following systemic therapy would not necessarily be expected to compromise the construct. Moreover, available evidence suggests that substantial tumour regression does not consistently translate into relief of SVC stenosis [30]. Late stent migration may be further mitigated both by device-specific features of the Wrapsody endoprosthesis (per manufacturer specifications: biocompatible ePTFE outer layer favouring stent graft embedding, nitinol skeleton allowing up to 25% oversizing, and softened scalloped end rows) [16–18] and, by design, by the geometric interlocking of the “double-D” configuration; consistently, no cases of stent migration have been reported in the principal published series of covered stenting for malignant SVC obstruction [9, 24, 26, 27].

This report is limited by the small sample size and heterogeneous follow-up; no follow-up CT documenting significant tumour regression was available at the time of writing, and the haemodynamic advantage hypothesized for the “double-D” configuration — supported by indirect arterial evidence [10–12, 31, 32] but not validated by flow modelling or imaging studies in the venous SVC setting, including in our own series — remains to be confirmed by future studies; furthermore, the anti-migration rationale has not been specifically validated in the setting of tumour regression after systemic therapy, as no dedicated imaging was available in any of our three patients; despite these limitations, CERAC appears to be a feasible and reproducible option for confluence-preserving endovascular reconstruction. Further studies are needed to assess long-term patency and refine patient selection.

## Conclusion

The CERAC technique represents a feasible endovascular option for reconstruction of the SVC–brachiocephalic vein confluence in selected cases of SVCS. By enabling confluence-preserving reconstruction with covered stents, CERAC may facilitate bilateral venous inflow while minimizing stent overlap. Further studies are required to assess long-term patency and to better define appropriate indications.

## Supplementary Information

Supplementary Material 1.

## Data Availability

Data sharing is not applicable to this article as the study reports a limited case series and no publicly available datasets were generated.

## Abbreviations

SVCS: Superior Vena Cava Syndrome
SVC: Superior vena cava
CERAC: Covered Endovascular Reconstruction of the Anonymous Confluence
CT: Computed tomography
CBCT: Cone beam computed tomography
US: Ultrasound
DAP: Dose area product
ESM: Electronic Supplementary Material
