# Supplementary material for: Covered Endovascular Reconstruction of the Anonymous Confluence (CERAC): a technical note describing a confluence-preserving endovascular technique
Source: CVIR Endovasc. 2026 Sep 15;9:119. doi: 10.1186/s42155-026-00765-w (PMC13578196; doi:10.1186/s42155-026-00765-w)
Supplement: Supplementary file 1 [file 42155_2026_765_MOESM1_ESM.docx]

# Covered Endovascular Reconstruction of the Anonymous Confluence (CERAC): a technical note describing a confluence-preserving endovascular technique

## Figures

**ESM-Fig. 1** **| CT nephrographic phase (MIP 10mm)**. Mixed germ cell mediastinal tumour in a young patient, with face and chest oedema and right arm swelling.
